# Reducing anxiety and enhancing physical performance by using an advanced version of EMDR: a pilot study

**DOI:** 10.1002/brb3.221

**Published:** 2014-02-12

**Authors:** Marco Rathschlag, Daniel Memmert

**Affiliations:** Institute of Cognitive and Team/Racket Sport Research, German Sport UniversityCologne, Germany

**Keywords:** Anxiety, EMDR, physical performance, treatment of anxiety, wingwave

## Abstract

**Background:**

The main aim of this pilot study was to investigate an advanced version of eye movement desensitization and reprocessing (EMDR) for reducing anxiety.

**Methods:**

Fifty participants were asked at two times of measurement (T1 and T2 with a rest of 4 weeks) to generate anxiety via the recall of autobiographical memories according to their anxiety. Furthermore, the participants were randomly assigned to an experimental group and a control group, and the experimental group received an intervention of 1–2 h with the advanced version of EMDR in order to their anxiety 2 weeks after T1. At T1 as well as T2, we measured the intensity of participants' anxiety with a Likert scale (LS) and collected participants' state (temporary) and trait (chronic) anxiety with the State-Trait Anxiety Inventory (STAI). In addition, we measured participants' physical performance in a test for the finger musculature under the induction of their anxiety.

**Results:**

The results showed that participant's ratings of their perceived intensity of anxiety (measured by a 9-point LS) and the state and trait anxiety decreased significantly in the experimental group but not in the control group from T1 to T2. Moreover, the physical performance under the induction of participants' anxiety increased significantly in the experimental group from T1 to T2 and there were no significant changes in the control group.

**Conclusions:**

The study could show that the advanced version of EMDR is an appropriate method to reduce anxiety.

## Introduction

Anxiety disorders are the most common mental illness in the United States, affecting around 40 million adults (ADAA 2013), with these individuals spending all together billions of dollars every year in treatments and remedies (Barlow 2002). Therefore, humans need methods that can help them to deal with their anxiety. Traditional methods, like for example, the cognitive-behavioral therapy (CBT), have been established as empirically supported treatments for anxiety disorders (e.g., Chambless and Ollendick 2001), however, they often require relative long periods of treatment: “The large majority of people who suffer from an anxiety disorder are able to reduce or eliminate their anxiety symptoms and return to normal functioning after several months of appropriate psychotherapy” (APA 2013). Hence, researchers are always looking for new methods which can also be successful in reducing anxiety symptoms using shorter periods of time.

The purpose of this paper was to investigate an advanced version of the technique of Eye movement desensitization and reprocessing (EMDR; Shapiro 1989) for the treatment of anxiety, the so-called wingwave method (Besser-Siegmund and Siegmund 2010, 2013). The inventors of this method affirm that the wingwave method is appropriate in reducing anxiety symptoms in only a few hours of intervention. The wingwave method utilizes the technique of EMDR (Shapiro 1989) as main intervention tool. EMDR was developed by Shapiro (1989) for the treatment of patients with posttraumatic stress disorder (PTSD) and has been empirically validated (Carlson et al. 1998; Marcus et al. 1997; Rothbaum 1997; Shapiro 1999). In EMDR treatment, the patient recalls trauma-related memories and while simultaneously attending to inner thoughts and sensory stimulation from a rhythmic, bilateral source. The sensory stimulus is most typically visual (hence “eye movement”), but can be auditory, tactile, or proprioceptive (Shapiro 2001). Furthermore, EMDR is not only used in the treatment of PTSD but also in the treatment of anxiety. There are several studies which could show that eye movements (EMDR) can decrease the emotional intensity of anxiety (Muris and Merckelbach 1997; De Jongh et al. 2002; Graham and Robinson 2007; Smeets et al. 2012). De Jongh and ten Broeke (2009) found that there is randomized outcome research for panic disorders (PD) and specific (i.e., spider) phobia, but not for other anxiety disorders (i.e., social phobia, obsessive-compulsive disorder, and general anxiety disorders [GAD]).

However, in addition to the intervention with EMDR, the wingwave method uses for the diagnosis of stress triggers and for evaluating the success of the treatment a muscle test named the Bi-Digital-O-Ring-Test (BDORT) originally developed by Omura (1985). The relationship between treatments for anxiety and muscle tension is until now poorly understood (Pluess et al. 2009). Barlow et al. (1984) investigated subjects diagnosed with general anxiety disorders (GAD) or PD, which were treated with CBT compared with a waiting-list control group. The authors could find a significant reduction in electromyography measures after the intervention in the CBT group. In the BDORT, which the wingwave method uses, a subject has to form a “ring” with the thumb and the index finger and the diagnostician tries to pry them apart. The idea of Besser-Siegmund and Siegmund (2010) is that subjects' strength of the finger musculature in the BDORT is different depending on which kind of emotion they self-generate and how good patients can deal with this emotion. Rathschlag and Memmert (2013) used an objective form of the BDORT and they found that subjects inducing self-generated emotions can generate a lower physical performance in the finger musculature when recalling anxiety and sadness in comparison to happiness or anger.

Wingwave combines BDORT and EMDR in a way that subjects only have to perform eye movements during anxiety-related recall of specific stressors when the subject cannot hold the “ring” of their thumb and their index finger together, when the diagnostician tries to pry them apart. That is, subjects' possible stress triggers will be tested with the BDORT and only the imagination of the triggers which lead to a decreased physical performance in the finger musculature will be treated with EMDR. Furthermore, Besser-Siegmund and Siegmund (2010, 2013) hypothesize that after a successful intervention with EMDR the physical performance in the BDORT is enhanced when participants are asked to self-generate their anxiety or specific stressors of their anxiety again. However, it has to be noticed that the underlying mechanism for the wingwave method are still poorly understood and thus, this study constitutes a first pilot study to investigate this method.

### The present research

The purpose of this pilot study was to contribute to research on treatment options for anxiety by exploring an advanced version of EMDR. In this study, the participants had to self-generate the emotion of anxiety by recalling an autobiographical memory. Furthermore, subjects were randomly assigned to either an experimental group or a control group. Between the two times of measurement (T1 and T2), where we checked participants' intensity of anxiety and their state and trait anxiety, the experimental group received an intervention of 1–2 h with respect to their anxiety with the wingwave method, whereas no intervention was employed to the control group. According to the ideas of Besser-Siegmund and Siegmund (2010), we hypothesized that the wingwave method will significantly decrease anxiety from T1 to T2 in the experimental group but not in the control group.

Furthermore, we checked for both times of measurement the strength in the finger musculature in our objective form of the BDORT, when participants self-generated their anxiety. One assumption of the advanced version of EMDR is that participants can deal with their anxiety after the intervention and the strength in the finger musculature will be enhanced when the anxiety will be induced once more. According to this assumption, we hypothesized that the strength in the finger musculature in the anxiety condition will increase significantly for the experimental group but not for the control group from T1 to T2.

## Method

### Participants

Twenty-two male and 28 female subjects (*M* = 23.30 years, SD = 2.19) with an age range between 20 and 32 years old participated in this study. They were recruited via announcements in local newspapers and at the campus of the local university. The subjects were randomly assigned to either an experimental group or a control group. Both groups were comparable with respect to age ([experimental group] EG: *M* = 24.10 years, SD = 2.05; [control group] CG: *M* = 22.50 years, SD = 2.34). The study was carried out in accordance with the Helsinki Declaration of 1975. Written informed consent was obtained from each participant prior to the experiment and the participants received no compensation for participation.

### Induction of anxiety

The emotion of anxiety was induced via the recall of a personal emotional episode which was connected to this emotion. Thus, participants had to imagine a very anxious moment in their lives where they could still feel this anxiety at the current time and were asked to relive this anxiety. There is already evidence that self-generating an emotion is an appropriate method to induce an emotional state-like anxiety (e.g., Damasio et al. 2000; Rathschlag and Memmert 2013). Previous results by Rathschlag and Memmert (2013) demonstrated that participants who self-generated the emotion of anxiety experienced significantly more anxiety in this condition compared with other emotions (e.g., happiness, anger, and sadness). These finding are in line with a lot of other studies (e.g., Lench and Levine 2005; Stopa and Waters 2005; Lench et al. 2011) and showed that anxiety can be generated in this way. In addition, participants were asked to recall the same personal emotional episode for both times of measurement.

### Measurement of the intensity of anxiety

We used a LS to assess the degree of which participants experienced the emotion of anxiety at the current time in relation to their anxious memory. Participants rated the emotional intensity of their anxiety, using a 9-point LS (emotional intensity: 1 = no anxiety to 9 = most anxiety).

### Measurement of state and trait anxiety

In addition, anxiety was recorded using the standardized State-Trait Anxiety Inventory (STAI; Laux et al. 1981). The STAI is a self-description questionnaire including two nondependent scales, the applied state-anxiety scale (STAI State) and the trait-anxiety scale (STAI Trait), each of them consisting of 20 items. The scale sum values range from 20 to 80. The STAI State assesses how respondents feel “right now, at this moment” (e.g., “I feel at ease;” “I feel upset”), and the STAI Trait targets how respondents “generally feel” (e.g., “I am a steady person;” “I lack self-confidence”). Respondents are asked to rate themselves on each item on the basis of a 4-point LS ranging from not at all to very much for the STAI State and from almost never to almost always for the STAI Trait. Measurements of the reliability of the STAI demonstrated excellent internal consistency (average *α* > 0.89), and the STAI Trait has an excellent test–retest reliability (average *r* = 0.88) at multiple time intervals (Barnes et al. 2002). Based on the nature of the construct, the temporal stability for the STAI State (average *r* = 0.70) is lower than for the STAI Trait. Furthermore, the STAI has evidenced adequate convergent and discriminant validity with other measures of state and trait anxiety (Spielberger 1983).

### Physical task

**Figure 1:**
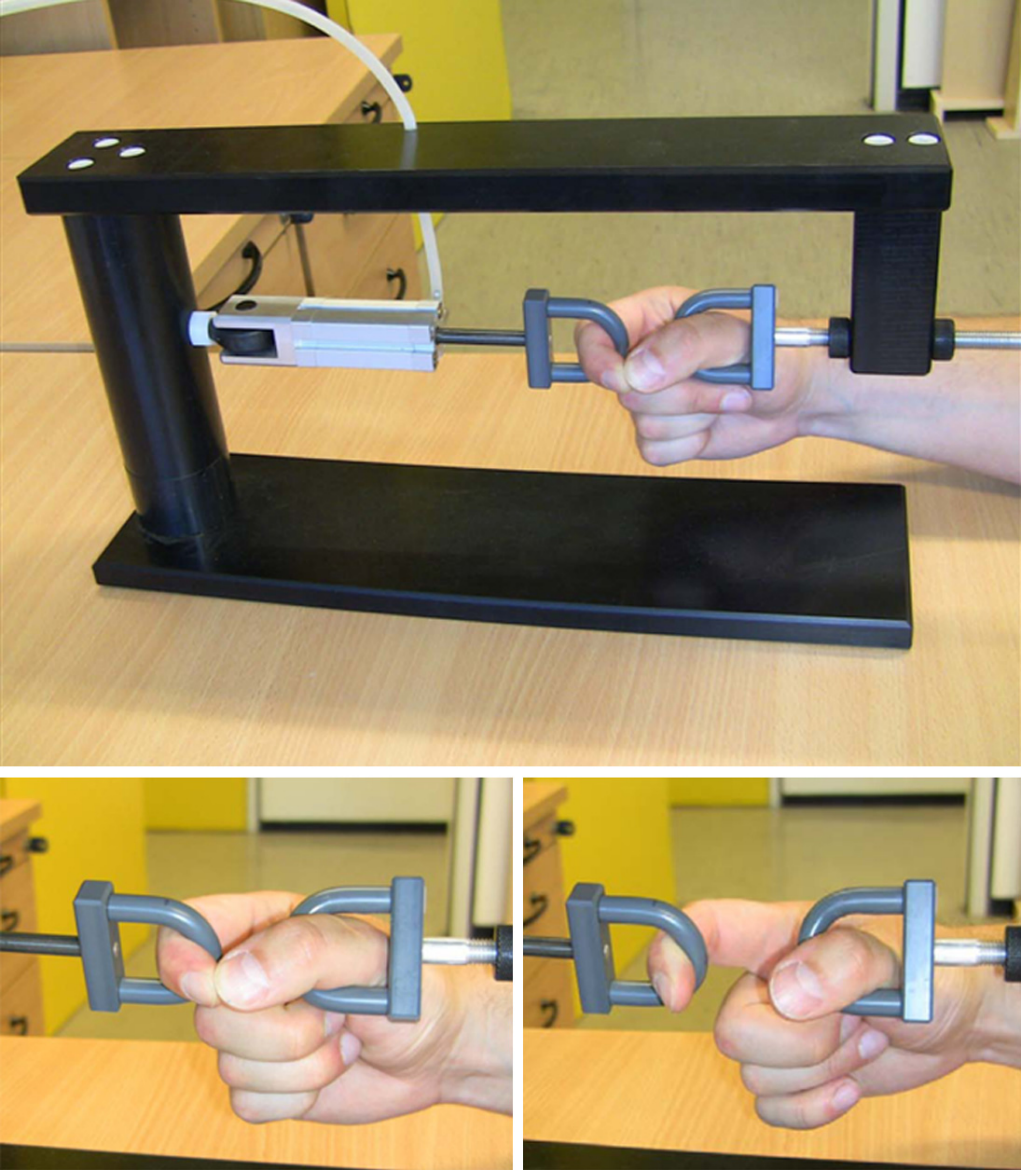
Experimental setup. Top: posture of arm, forearm, and especially of index and thumb during the task. Bottom left: posture of index and thumb rated as “closed ring” coded with “2”. Bottom right: posture of index and thumb rated as “unclosed ring” coded with a “1”.

We used a machine (see Fig. 1) that represents an objective measurement of the BDORT, developed by Omura (1985), to measure the strength of the finger musculature. This machine was already utilized by Rathschlag and Memmert (2013) and the authors could show that the machine is an objective and reliable measurement for the strength of the finger musculature. The machine generated a pulling force that separates the index finger and the thumb when they touch each other to form a ring and the strength of the puling force could be controlled by a regulator. We first started to investigate participant's maximal strength using the one repetition maximum which was defined as the highest pulling force at which participants can still hold the ring of index and thumb together. Therefore, the strength of the pulling force was added in small increments (0.5–1.0 bar), with a resting period of 30 sec between measurements, until the subject could no longer hold the ring of index finger and thumb together. All measurements under the emotion of anxiety were tested at 90% of participants' individual maximum voluntary contraction (MVC). To analyze the measurements, we filmed participants' hands by a digital camera and the film material was observed by three raters who had to decide independently whether the ring of index finger and thumb was open or closed. The raters were neither informed about the purpose of this study, nor which emotion participants had to induce. Further, the raters were not informed about the allocation of the participants in two different groups (experimental group vs. control group). The coding system was the following: 1.0 = ”unclosed ring”, 1.3 = ”approximately unclosed ring”, 1.7 = ”approximately closed ring”, 2.0 = ”closed ring”. After we assessed interrater-reliability of the three different subjective strength ratings, the mean of the three rater judgments (mean of the six measurements under the emotion of anxiety) was used for analysis.

### Procedure

Participants were tested individually and were instructed that we want to investigate a new method for the treatment of anxiety. Further, participants were informed that they have to fill out three questionnaires for anxiety measurement and that they will be allocated to one of two groups (experimental group vs. control group). At T1, after providing demographic information and written consent, participants filled out the STAI (Laux et al. 1981). Subsequently, participants were familiarized with the machine for the objective measurement of the BDORT and we tested the individual MVC of the participants. Following this, participants were asked to think of a situation in which they had experienced their anxiety. When participants confirmed that they had a situation in their mind, they had one minute to self-generate this emotion and to indicate the intensity of anxiety on the corresponding LS. Immediately afterwards, participants put their thumb and index finger in the machine for the objective measurement of the BDORT and performed six measurements of the force of the finger musculature (90% MVC) under the emotion of anxiety, with breaks of 30 sec in between each of the six trials. The moment in which the machine generated the pulling force was announced by an acoustic signal 3 sec in advance. From that moment on, participants were asked to hold the ring of index finger and thumb together with their maximum force and go on with self-generating the emotion. After one trial, participants were asked to relax their fingers in the machine until the next acoustic signal but go on with self-generating their anxiety in the rest intervals between the trials. Participants completed six trials under the emotion of anxiety.

The participants had been randomly assigned to an experimental group or a control group after T1. Two weeks after T1, the experimental group received only one single intervention (about 1–2 h) with the wingwave method by a qualified wingwave coach and the control group received no intervention. Further 2 weeks later, at T2, participants were asked to fill out the same questionnaires and to perform the same physical task as in T1. The 25 participants in the experimental group were randomly allocated to five different qualified wingwave coaches, who were comparable in relation to years of expertise with the wingwave method, and thus, each wingwave coach conducted an intervention with this method with five participants. The procedure for T2 was the same as described above for T1.

### Data analysis

All consent forms containing identifiable information were kept completely confidentially and separately from the completed questionnaires, which were only identifiable by an allocated ID number. First, we assessed participants' intensity of anxiety (measured by a 9-point LS ranging from no anxiety to most anxiety) in relation to their anxious memory in both groups and for both times of measurement. Therefore, data were analyzed using a 2 (group: experimental group vs. control group) × 2 (time of measurement: T1 vs. T2) analysis of variance (ANOVA) with repeated measures on the second factor. Second, after checking the interrater-reliability for the three different strength ratings, by calculating intraclass correlation coefficients (ICC; Shrout and Fleiss 1979), we compared both groups referred to their average strength in the BDORT (after inducing their anxiety) for both times of measurement. The data were analyzed using a 2 (group: experimental group vs. control group) × 2 (time of measurement: T1 vs. T2) ANOVA with repeated measures on the second factor. Third, we compared the experimental group and the control group in relation to the data in the STAI-G, divided into STAI-G-State and STAI-G-Trait for both times of measurement. Hence, data were analyzed using two 2 (group: experimental group vs. control group) × 2 (time of measurement: T1 vs. T2) ANOVAs with repeated measures on the second factor.

## Results

### Intensity of anxiety

**Figure 2:**
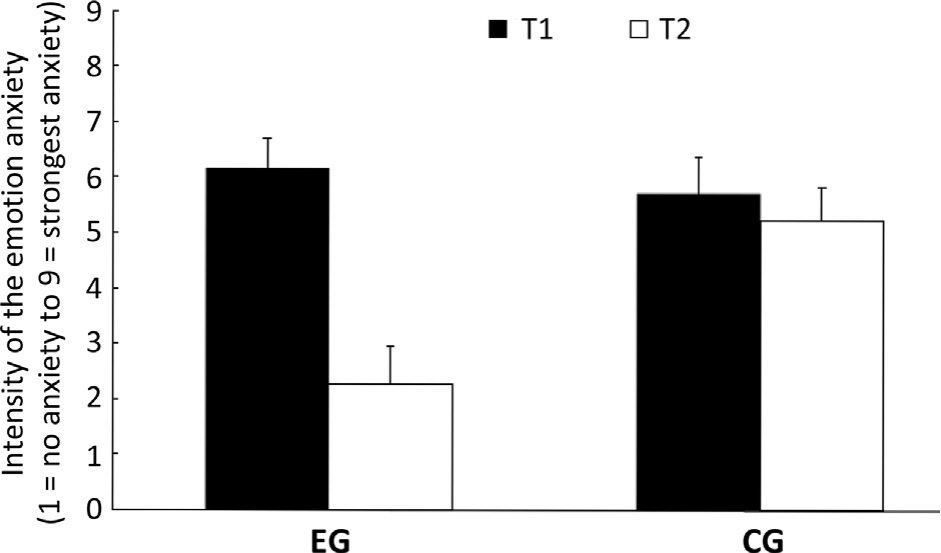
Likert Scale (LS) for the intensity of anxiety in the experimental group (EG) and the control group (CG) for time of measurement 1 (T1) and time of measurement 2 (T2).

The ANOVA did not reveal an main effect for time of measurement (*F*(1, 47) = 3844) and for group (*F*(1, 47) = 0.472). However, there was a significant interaction between time of measurement and group (*F*(1, 47) = 9.26, *P* < 0.008, *η*² = 0.16). For T1, the mean values of anxiety did not differ significantly between both groups. However, the interaction indicated that the mean values of anxiety decreased in the experimental group from the first to the second time of measurement and the mean values of anxiety in the anxiety condition were as far as possible unchanged (see Fig. 2).

### Physical task

**Figure 3:**
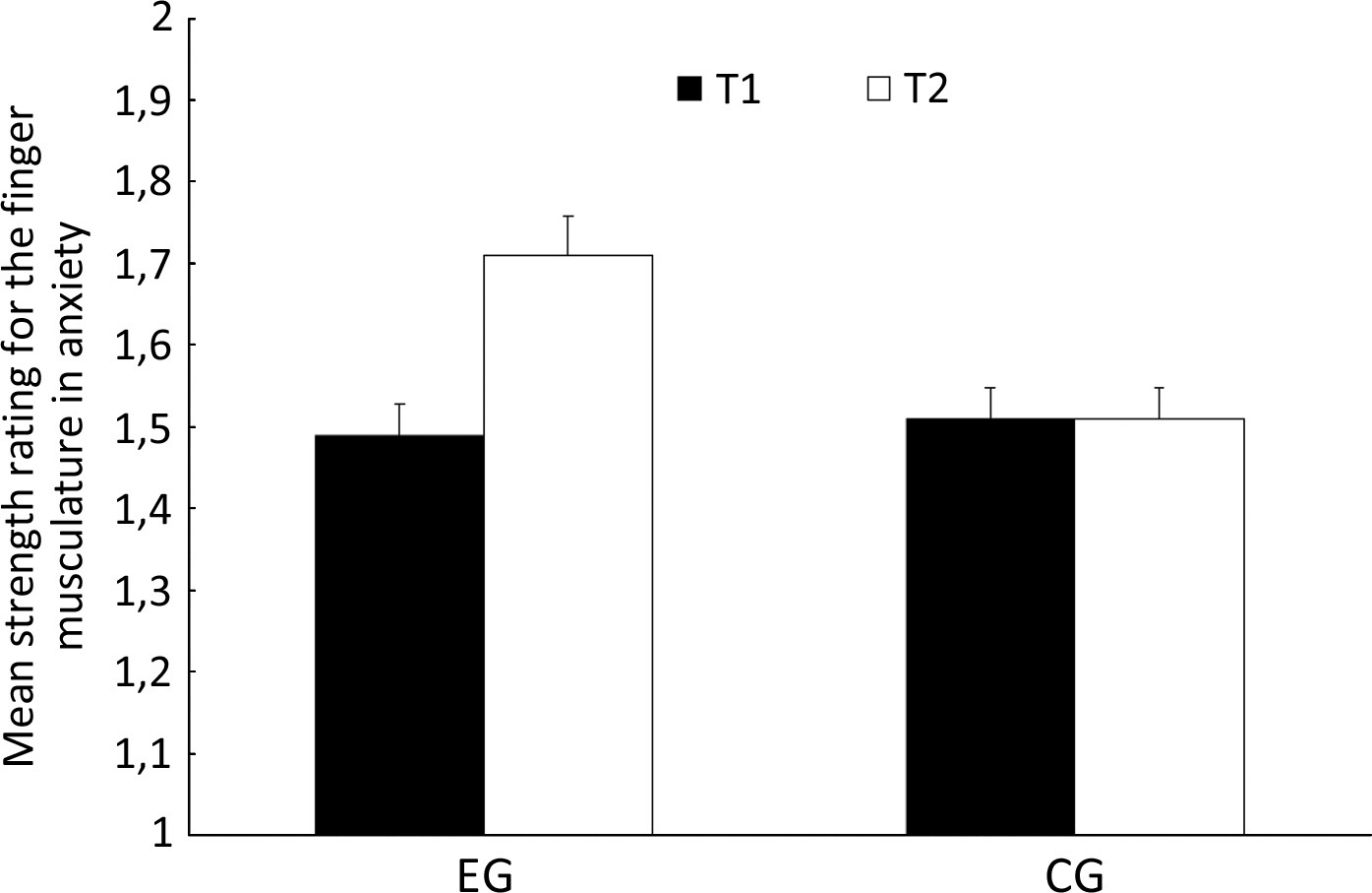
Mean strength rating and standard errors for the emotion anxiety in the experimental group (EG) and in the control group (CG) for time of measurement 1 (T1) and time of measurement 2 (T2).

First, the interrater-reliability coefficients were acceptable for all judges (ranging from 0.90 to 0.96 and averaging 0.93) for both times of measurement. The subsequent 2 (group: experimental group vs. control group) × 2 (time of measurement: T1 vs. T2) ANOVA yields a main effect for time of measurement (*F*(1, 48) = 13.44, *P* < 0.001, *η*² = 0.21) but not for group (*F*(1, 48) = 3.20). In addition, we found a significant interaction between group and time of measurement (*F*(1, 48) = 12.96, *P* < 0.001, *η*² = 0.21). Figure 3 shows that the mean data for strength (after the anxiety induction) increased in the experimental group from T1 to T2 and the strength in the control group was as far as possible unchanged from T1 to T2.

### STAI-G-State

**Figure 4:**
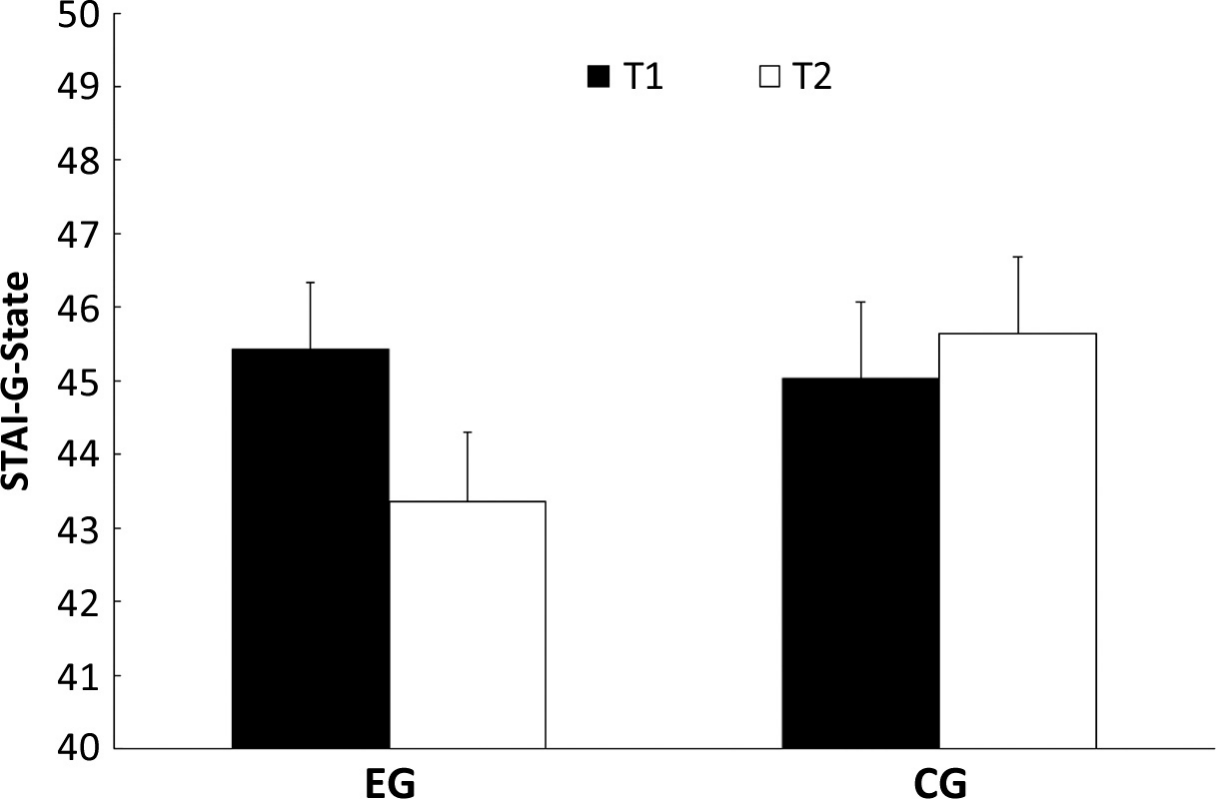
Mean and standard errors in the STAI-G-State questionnaire for the experimental group (EG) and the control group (CG) for time of measurement 1 (T1) and time of measurement 2 (T2).

The ANOVA revealed no significant main effect for group (*F*(1, 48) = 1.74) or for time of measurement (*F*(1, 48) = 0.54). However, we found a significant interaction between group and time of measurement (*F*(1, 48) = 5.73, *P* < 0.022). The mean data in the experimental group decreased from the first time of measurement to the second time of measurement, while the data in the control group increased from the first time of measurement to the second time of measurement. Figure 4 shows the mean data in the STAI-G-State questionnaire in the in the experimental group and the control group divided into the two times of measurement.

### STAI-G-Trait

**Figure 5:**
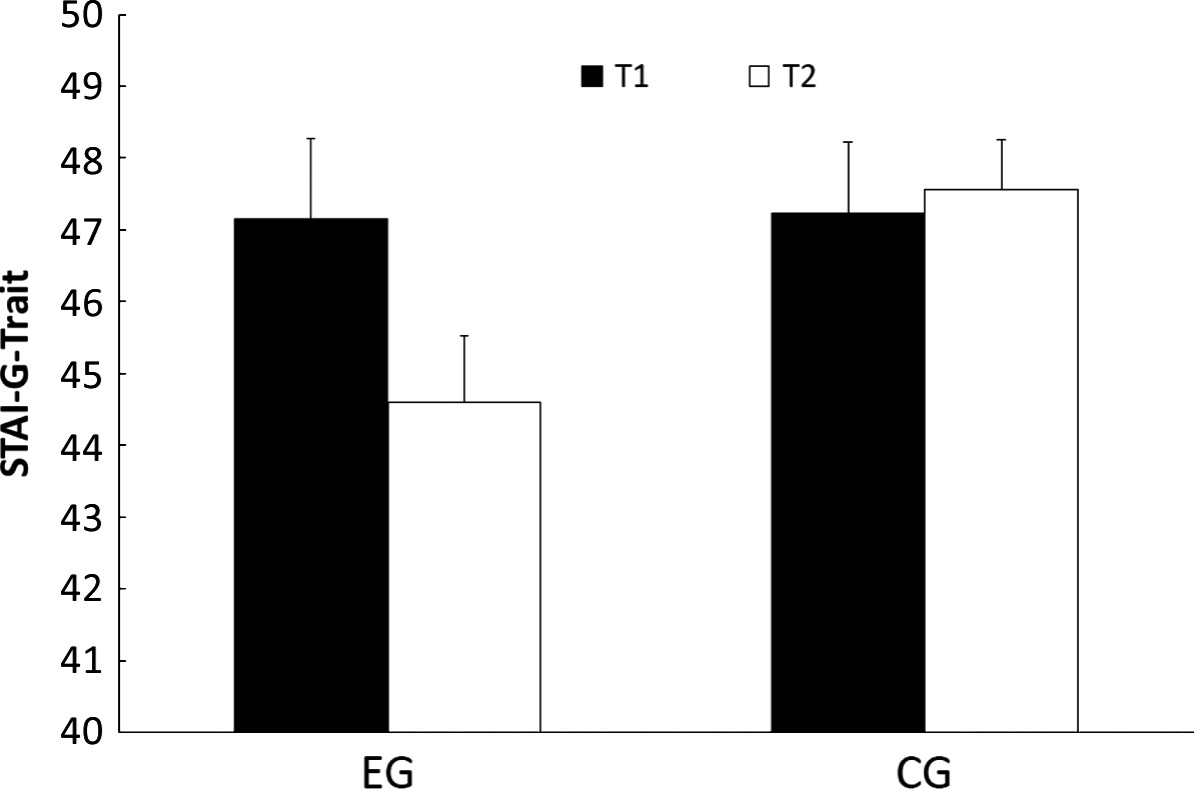
Mean and standard errors in the STAI-G-Trait questionnaire for the experimental group (EG) and the control group (CG) for time of measurement 1 (T1) and time of measurement 2 (T2).

The ANOVA revealed no significant main effect for group (*F*(1, 48) = 1.72) or for time of measurement (*F*(1, 48) = 2.85). The interaction between group and time of measurement (*F*(1, 48) = 4.76, *P* < 0.035) was significant. The mean data in the experimental group decreased from the T1 to T2, while the data in the control group increased from T1 to T2. Figure 5 shows the mean data in the STAI-G-Trait questionnaire in the experimental group and the control group divided into the two times of measurement.

## Discussion

The main aim of this pilot study was to investigate for the first time the efficacy of an advanced version of EMDR according an intervention of anxiety. Between two times of measurement, the experimental group received an intervention of 1–2 h with respect to their anxiety with the wingwave method, whereas no intervention was employed to the control group. All participants were asked at both times of measurement to self-generate the emotion of anxiety via the recall of an autobiographical memory. Previous studies have already demonstrated that the self-generation of an emotion is an appropriate way to induce an emotion like anxiety (e.g., Damasio et al. 2000; Rathschlag and Memmert 2013). We investigated the intensity of anxiety, the physical performance under the emotion of anxiety and the state and trait anxiety with the STAI (Laux et al. 1981) for both groups and for both times of measurement.

First of all, the results demonstrated that the intensity of anxiety did not differ at T1 between both groups and decreased from T1 to T2 in the experimental group but not in the control group. In this respect, we provided evidence for our hypothesis that the wingwave method can help to decrease the intensity of anxiety concerning to the respective anxious memories and their recall, and to make the recall more difficult. This result is in line with several studies that have found that making eye movements (EMDR) while retrieving visual images of negative autobiographical memories reduces their vividness and emotional intensity (e.g., Smeets et al. 2012). In addition, Engelhard et al. (2011) could show that eye movements (EMDR) can also reduce the vividness and emotional intensity of recurrent, intrusive visual images about potential future catastrophes (“flashforwards”).

Secondly of all, we were interested to see if the strength in the physical task for the finger musculature, when people self-generate their anxiety, will change from T1 to T2 in the respective groups. The results provided primary evidence that the wingwave method is able to enhance participants' strength if inducing an anxious memory. Congruent with our hypothesis, data demonstrated that the strength in the experimental group was significantly enhanced from T1 to T2, and there were no significant differences in the control group. Thus, it seems that the wingwave method is helpful in enhancing physical strength in a task for the finger musculature when participants self-generate the emotion of anxiety. However, the rationale for this mechanism is not clear at the current time, which is a limitation of this study. Further studies have to find out a biological explanation for this result. One possible explanation might be that the participants can better deal with their anxiety after the intervention. Thus, the anxious memory is afterwards not more connected to feelings like to be paralyzed with anxiety, which might be the cause for a reduced strength prior to the intervention.

Third of all, we also checked participant's state and trait anxiety which were recorded with the STAI (Laux et al. 1981) and consisted of the following two nondependent scales: The state-anxiety scale (STAI-G Form X1) and the trait-anxiety scale (STAI-G Form X2). The results of the ANOVAS for both scales showed a significant interaction between the group and the time of measurement. The values in the experimental group decreased significantly from T1 to T2 in both scales and the values in the control group were unchanged from T1 to T2. Thus, the wingwave method seems to be a very powerful method to reduce state anxiety as well as trait anxiety. Similar results could be found by Graham and Robinson (2007) who found that EMDR can decrease significantly state anxiety in swimmers who had experienced a traumatic swimming event. This result is also in line with De Jongh et al. (2002) who found that EMDR is an effective treatment alternative for anxiety and can reduce this emotion. To the best of our knowledge, we could show for the first time that a technique like the wingwave method which uses EMDR as main intervention tool cannot only decrease participants' state anxiety but also participants' trait anxiety.

As a first study limitation, the induction of anxiety in our pilot study has his weaknesses. We only used subjective measures of anxiety by using our different scales. Future studies can also include objective measures of anxiety (e.g., galvanic skin response, heart rate or an electromyogram) to increase the validity of the study.

As a second study limitation, we have to say that in the present study, we investigated the possibility to reduce anxiety in general using the wingwave method. Thus, one recommendation for future research would be to investigate the effectiveness of the wingwave method in the treatment of discrete anxiety disorders, like the general anxiety disorder, phobias, or PD. As an important study limitation we have to say that we only compared two groups in the present study: an experimental group and a control group. Given a large placebo response in several samples, it could be that our results are only the consequences of a placebo effect. Thus, future studies should also add a placebo group to the experimental design. To compare the effectiveness of the wingwave method to CBT for example, researchers could further add another group which gets an intervention with the CBT. A comparison of EMDR and CBT in the treatment of PD was already done by Faretta (2012) and the results showed that both treatments are effective for the resolution of a PD. However, EMDR treatment seems to have a faster progress in symptom reduction which is maintained over time. Another comparison could be conducted between EMDR and wingwave in the treatment of anxiety and analyses can potentially show if the wingwave method is an improved alternative to EMDR. Furthermore, future research could check, if there are time-dependent effects of the method. In the present study, participants received an intervention with the wingwave method 2 weeks after the first time of measurement and another 2 weeks later, participants completed the second time of measurement. Thus, it may be interesting to find out how stable the present results are over a longer period of time.

To the best of our knowledge, the present study was the first one to investigate the effects of the wingwave method in reducing anxiety. The results from this pilot study seem promising to help people in the future to decrease rapidly their anxiety. We hope that this study will help to inform and motivate future research to further investigate this new method in the treatment of anxiety.
